# The conservation status and population decline of the African penguin deconstructed in space and time

**DOI:** 10.1002/ece3.6554

**Published:** 2020-07-09

**Authors:** Richard B. Sherley, Robert J. M. Crawford, Andrew D. de Blocq, Bruce M. Dyer, Deon Geldenhuys, Christina Hagen, Jessica Kemper, Azwianewi B. Makhado, Lorien Pichegru, Desmond Tom, Leshia Upfold, Johan Visagie, Lauren J. Waller, Henning Winker

**Affiliations:** ^1^ Centre for Ecology and Conservation College of Life and Environmental Sciences University of Exeter Penryn UK; ^2^ FitzPatrick Institute of African Ornithology DST‐NRF Centre of Excellence University of Cape Town Cape Town South Africa; ^3^ Department of Environment, Forestry and Fisheries (DEFF) Cape Town South Africa; ^4^ Seabird Conservation Programme BirdLife South Africa Cape Town South Africa; ^5^ CapeNature PGWC Shared Services Centre Bridgetown South Africa; ^6^ African Penguin Conservation Project Lüderitz Namibia; ^7^ DST/NRF Centre of Excellence at the FitzPatrick Institute of African Ornithology Institute for Coastal and Marine Research and Department of Zoology Nelson Mandela University Port Elizabeth South Africa; ^8^ Ministry of Fisheries and Marine Resources Lüderitz Namibia; ^9^ Southern African Foundation for the Conservation of Coastal Birds (SANCCOB) Cape Town South Africa; ^10^ Department of Biodiversity and Conservation Biology University of the Western Cape Bellville South Africa; ^11^ Joint Research Centre of the European Commission Ispra Italy

**Keywords:** Bayesian state‐space model, Benguela ecosystem, extinction risk, IUCN Red List assessment, population dynamics, seabird conservation

## Abstract

Understanding changes in abundance is crucial for conservation, but population growth rates often vary over space and time. We use 40 years of count data (1979–2019) and Bayesian state‐space models to assess the African penguin *Spheniscus demersus* population under IUCN Red List Criterion A. We deconstruct the overall decline in time and space to identify where urgent conservation action is needed. The global African penguin population met the threshold for *Endangered* with a high probability (97%), having declined by almost 65% since 1989. An historical low of ~17,700 pairs bred in 2019. Annual changes were faster in the South African population (−4.2%, highest posterior density interval, HPDI: −7.8 to −0.6%) than the Namibian one (−0.3%, HPDI: −3.3 to +2.6%), and since 1999 were almost −10% at South African colonies north of Cape Town. Over the 40‐year period, the Eastern Cape colonies went from holding ~25% of the total penguin population to ~40% as numbers decreased more rapidly elsewhere. These changes coincided with an altered abundance and availability of the main prey of African penguins. Our results underline the dynamic nature of population declines in space as well as time and highlight which penguin colonies require urgent conservation attention.

## Graphical abstract

Understanding changes in abundance over space and time is crucial for conservation. We use 40 years of count data and Bayesian state‐space models to deconstruct the decline of the African penguin population (almost 65% since 1989) in time and space. Our results underline the dynamic nature of population declines and highlight areas in need of urgent conservation attention, such as the South African colonies north of Cape Town that have declined at nearly 10% per annum since 1999.

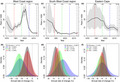

## 1 INTRODUCTION

Seabirds are considered to be the most threatened group of birds in the world (Croxall et al., 2012); globally their populations may have declined by >70% since 1950 (Paleczny, Hammill, Karpouzi, & Pauly, 2015). Seabirds face a number of threats both on land in their colonies, like invasive non‐native species and disturbance, and in the oceans, such as bycatch and competition with fisheries (Dias et al., 2019). Seven seabird species breed only within the influence of the Benguela upwelling ecosystem of Southern Africa (Angola, Namibia, and South Africa). Five of these endemics are listed in a threatened category (Vulnerable or worse) on the International Union for Conservation of Nature (IUCN) Red List, including the African penguin *Spheniscus demersus*, which was first listed as Endangered in 2010 (Crawford et al., 2011).

**FIGURE 1:**
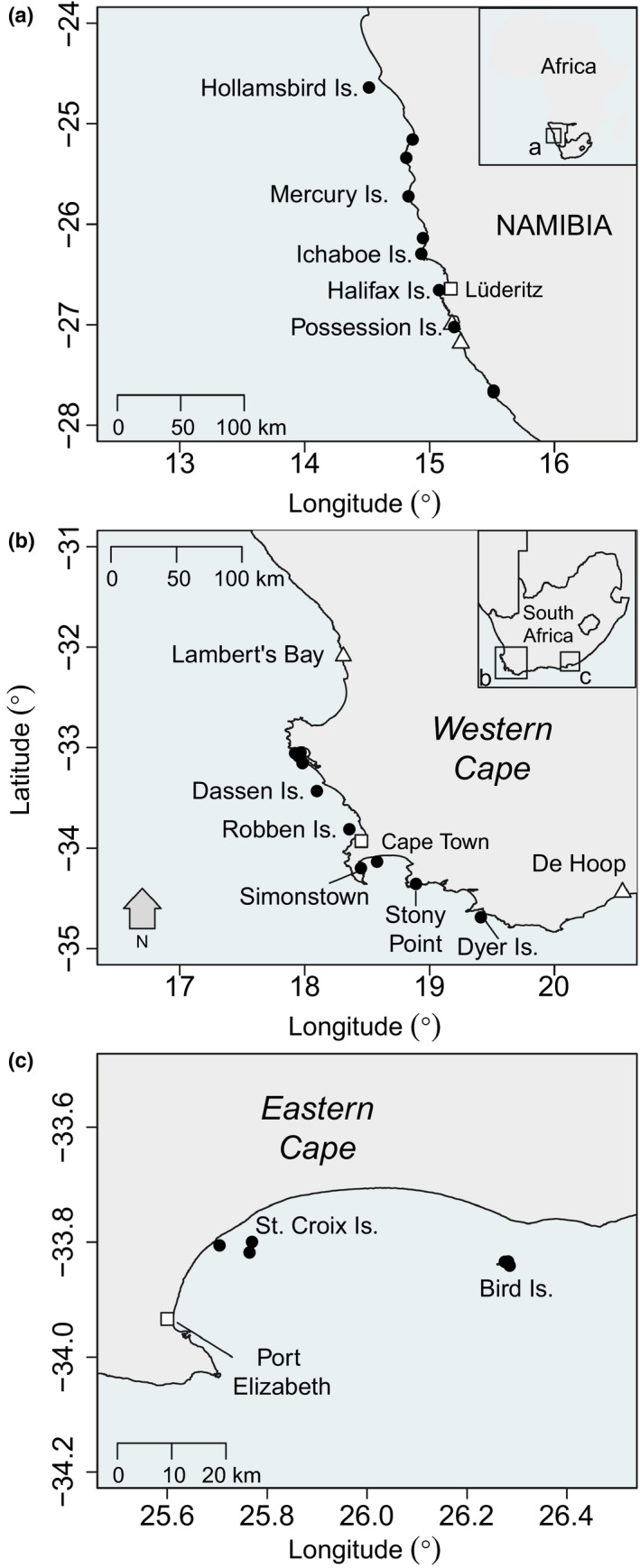
The 28 extant (●) and 4 extinct (△) breeding colonies of the African penguin in South Africa and Namibia. Colonies mentioned in the text are named, as are the major towns and cities (□) in each region

The African penguin breeds, or has bred, at 32 island and mainland colonies between central Namibia (Hollamsbird Island) and South Africa's Eastern Cape province (Bird Island; Figure 1) (Crawford, Kemper, & Underhill, 2013). The breeding colonies are clustered in three core groups, Namibia, South Africa's Western Cape, and South Africa's Eastern Cape, each separated from another by c. 600 km (Figure 1). Although the total population at the turn of the 20th century is not known, there may have been as many as 1.5–3.0 million individuals across the species' range and 0.3 million pairs on Dassen Island alone (Crawford, Underhill, Upfold, & Dyer, 2007; Frost, Siegfried, & Cooper, 1976; Shannon & Crawford, 1999). By 1956, only an estimated 0.3 million individuals remained, and the population has more or less declined consistently since then, apart from a period in the late 1990s and early 2000s when numbers in the Western Cape briefly recovered (Crawford et al., 2011). This population change since the 1950s has been attributed to a number of top–down and bottom–up processes, including historical egg collecting and guano scraping, changes in the abundance and distribution of their main prey (sardine *Sardinops sagax* and anchovy *Engraulis encrasicolus*), pollution, habitat loss and modification, predation on land in their colonies and at sea, competition with fisheries, and climate change (Crawford, 2007; Crawford, Makhado, & Oosthuizen, 2018; Frost et al., 1976; Sherley et al., 2017).

African penguin breeding populations have been counted at all major colonies in South Africa since 1979 and at the four major colonies in Namibia since 1985 (Crawford et al., 2013). Here, we use these count data and a generalized Bayesian state‐space tool for estimating extinction risk under IUCN Red List Criterion A (Just Another Red List Assessment [JARA], Sherley, Winker, et al., 2020; Winker, Pacoureau, & Sherley, 2020) to assess the current status of the African penguin population at a global scale. We then deconstruct the overall decline in time and space to identify the regional populations most in need of urgent conservation action. Finally, we review the threats faced by the species and identify interventions needed to secure the species' conservation in light of our findings.

## 2 MATERIALS AND METHODS

### 2.1 Penguin count data

In South Africa, the number of occupied nest sites of African penguins was counted at most extant breeding colonies sporadically between 1979 and 1991 and annually thereafter (Crawford et al., 2011; Shelton, Crawford, Cooper, & Brooke, 1984). We used counts from 18 localities where penguins bred in South Africa for more than 5 of the 41 years from 1979 to 2019 (Figure S1, Appendix 1). Of a possible 738 annual counts, 472 were completed and 265 were not made. In Namibia, counts are made monthly, with the highest count taken to represent the annual estimate. This process was undertaken nearly annually between 1985 and 2019 at the four major colonies that constitute >95% of the breeding population in that country: Mercury Island, Ichaboe Island, Halifax Island, and Possession Island (Kemper, 2015; Figure S2, Appendix 1).

The methods used to count the numbers of occupied nest sites of African penguins have been outlined in detail elsewhere (Crawford et al., 2011; Shelton et al., 1984). Briefly, counts were undertaken by teams of people walking through a penguin colony and counting occupied nest sites. Larger colonies were broken down into predefined census areas, each of which was counted separately. Counts in South Africa were predominately made between February and September each year, those in Namibia were made monthly (Crawford et al., 2011, 2013). At some small and difficult to access localities, counts made outside the main breeding seasons were used if no other count was available for that year. Where more than one count was made at a locality in a year, the highest count was taken to represent the number of pairs breeding that year (Crawford et al., 2011). In Namibia, an occupied site was a penguin nest containing fresh eggs or chicks. In South Africa, an occupied site was considered active if it contained fresh eggs or chicks, or was defended by an adult penguin (or pair of penguins) that were not in molt, and considered potential if it was not active but showed recent signs of use, for example, the presence of substantial fresh guano or nesting material, the recent excavation of sand from a burrow nest, the presence of many penguin footprints in its vicinity, or a combination of these factors. Breeding by African penguins is not always synchronous (Crawford, Shannon, & Whittington, 1999), so potential nests were counted as they may be occupied by pairs that have recently finished breeding or that are about to breed (Crawford et al., 2011). Groups of unguarded chicks (crèches) were divided by two to estimate the number of nest sites they represented (mean clutch size is ~1.8 eggs; Crawford et al., 1999; Shannon & Crawford, 1999), with remainders taken to represent an additional site, for example crèches of five and six chicks would both be taken to represent three nests (Shelton et al., 1984).

### 2.2 Generation length

The generation length (*G*) for African penguins was calculated as:

(1) $$G=A+\frac{1}{\left(1‐\phi_{A}\right)}$$

where *A* is age of first breeding and $\phi_{A}$ is adult survival (BirdLife International, 2000). The IUCN Red List guidelines state “where generation length varies under threat… the more natural, that is predisturbance, generation length should be used” (IUCN Standards & Petitions Subcommittee, 2019). Accordingly, we used $\phi_{A}$ = 0.81 based on capture‐mark‐recapture studies at Robben and Dassen Island between 1989 and 1998 (Whittington, 2002) and between 1994/95 and 1998/99 (Sherley, Abadi, et al., 2014). African penguins can breed for the first time at between 4 and 6 years of age (Whittington, Klages, Crawford, Wolfaardt, & Kemper, 2005). Together these values yield generation length estimates of between 9.2 and 11.2 years. The previous assessment of African penguins used *G* = 10 years, and this value has been supported by a recent meta‐analysis of generation lengths in birds (Bird et al., 2020). Thus, we use *G* = 10 years here for consistency.

### 2.3 JARA state‐space framework

To determine the trend and rate of change of the African penguin population, we used JARA, a generalized Bayesian state‐space tool for IUCN Red List assessments under Criterion A (Winker et al., 2020) that has been applied recently to the Cape gannet *Morus capensis* (Sherley et al., 2019) and several pelagic sharks (Sherley, Winker, et al., 2020). JARA assumes that the underlying trend in the population (*I_t_*) follows a conventional exponential growth model (Kéry & Schaub, 2012):

(2) $$I_{t+1}=I_{t}\lambda_{t}$$

where $\lambda_{t}$ is the growth rate in year *t*. JARA includes an option to include a carrying capacity (which can be switched on and off by the user) to constrain population growth to be logistic rather than exponential. However, we did not use it here as it predominately applies to projections (which we do not make) and because the penguin population is well below its former carrying capacity (Crawford et al., 2007). On the log‐scale, the process model was:

(3) $$\mu_{t+1,i}=\mu_{t,i}+r_{t,i}$$

where $\mu_{t,i}=\log\left(I_{t,i}\right)$ and $r_{t,i}=\log\left(\lambda_{t,i}\right)$ are the year‐to‐year variation in log‐growth rates at breeding colony *i* that is assumed to vary around ${\bar{r}}_{i}$—the underlying mean rate of change for the colony—but with an estimable process variance $\sigma_{\eta }^{2}$ that was common to all colonies $r_{t,i}∼\text{Normal(}{\bar{r}}_{i},\sigma_{\eta }^{2})$. Because the process error was log‐normally distributed, we adjusted $r_{t,i}$ for log‐normal bias by subtracting half the variance (otherwise the stochastic log‐normal error induces a small positive bias) at each time step: ${\bar{r}}_{i}+r_{t,i}‐\left(0.5*\sigma_{\eta }^{2}\right)$ following Methot and Taylor (2011). The corresponding observation equation was:

(4) $$\log\left(y_{t,i}\right)=\mu_{t,i}+\epsilon_{t,i}$$

where $y_{t,i}$ is the number of pairs breeding in year *t* and $\epsilon_{t,i}$ is the observation residual for year *t* at breeding colony *i*. The residual error is assumed to be normally distributed on the log‐scale $\varepsilon_{t,i}∼\text{Normal}\left(0,\sigma_{\varepsilon }^{2}\right)$ as a function of a common observation variance $\sigma_{\varepsilon }^{2}$, which is itself separated into two components: (1) a fixed input variance $\sigma_{\mathrm{fix}}^{2}$ and (2) an estimable variance $\sigma_{\mathrm{est}}^{2}$. Adding a fixed observation error is common practice to account for additional sampling error associated with abundance indices (Maunder & Piner, 2017); this informs the estimate of the process variance as a portion of total variance is assigned a priori to the observation variance (Winker, Carvalho, & Kapur, 2018) and setting a minimum plausible observation error in this way helps to increase model stability and convergence of state‐space models (Auger‐Méthé et al., 2016). Total observation errors for abundance indices are typically in the range 0.1–0.4 (Francis, Hurst, & Renwick, 2003). Here, we set $\sigma_{\mathrm{fix}}^{2}$ = 0.15^2^ = 0.0225 for all models to aid convergence for the estimates of ${\bar{r}}_{i}$, particularly at colonies that show both strong increases and decreases across the time‐series (e.g., Robben Island, Figure S1, Appendix 1).

The estimated total population ${\hat{I}}_{p,t}$ for year *t* was computed from the sum of all individual colony trajectory posteriors after correcting them for the log‐normal bias by subtracting half the variance (Che‐Castaldo et al., 2017; Methot & Taylor, 2011):

(5) $${\hat{I}}_{p,t}=\sum_{i}\exp\left(\mu_{t,i}‐0.5*\sigma_{\mu_{t,i}}^{2}\right)$$

The skew in log‐normal distributions usually leads to the mean being biased high and while the median is generally a better measure of central tendency in skewed distributions than the mean, the sum of multiple log‐normal distributions is not log‐normally distributed itself (Dufresne, 2004). The result is that while the median represents an unbiased estimate of the population at each individual colony, the sum of the medians from several log‐normal distributed random variables does not equal the median of the sums. Thus, the posterior median of ${\hat{I}}_{p,t}$ would yield biologically unreasonable population estimates, particularly when population counts were large (Che‐Castaldo et al., 2017). Our corrected mean, however, had a mean bias of <0.001% (range = −0.05 to 0.05) relative to the sum of the medians (Figure S3, Appendix 1).

The overall change (%) in the population over 30 years (3G) at each colony was calculated from the posteriors of the estimated population trajectory (${\hat{I}}_{p,t}$) directly as the difference between the median of three years around the final observed data point *T* (median of ${\hat{I}}_{p,T}$, ${\hat{I}}_{p,T‐1}$ and ${\hat{I}}_{p,T+1}$), and a three‐year median around year T‐(3G). The year T+1 was always projected to obtain a three‐year median around *T* to reduce the influence of short‐term fluctuations (Froese, Demirel, Coro, Kleisner, & Winker, 2017). We present the posterior medians and probability distributions for the overall change in numbers over 30 years (3G) and present the posterior medians and probability distributions for the annual rates of change (${\bar{r}}_{i}$) converted to a percentage: $(\exp\left({\bar{r}}_{i}\right)‐1)\times 100$.

### 2.4 Regional variation in conservation status and decline rates

We first fit JARA simultaneously to the data from all 22 breeding colonies (18 in South Africa and 4 in Namibia) to determine the global trend, conservation status, and rates of decline for the African penguin over the last three generation lengths (3G or 30 years). Thereafter, we subset the data and refit JARA to (a) the four Namibian colonies only to determine the trend, national status, and rates of decline for Namibia; (b) the 18 South African colonies only, to give a perspective on the South African population. Then, to examine regional differences within South Africa, we further subset the data into (c) a West Coast region, in which we considered the seven South African colonies in the Western Cape that are north of Cape Town (Lambert's Bay to Robben Island, Figure 1); (d) a South‐West Coast region, which included the five Western Cape colonies south and east of Cape Town (Simonstown to Dyer Island, Figure 1); and (e) the six Eastern Cape colonies (Figure 1). To help model convergence, we used a value of 0.1 for the first year in time‐series for colonies that were not yet occupied in 1979 (Robben Island, Stony Point and Simonstown) and for the last year at Lambert's Bay as that colony went extinct in 2006 (Figure S1, Appendix 1).

### 2.5 Bayesian implementation

We implemented JARA in JAGS (v. 4.3.0) (Plummer, 2003) via the “jagsUI” library (v. 1.5.1) (Kellner, 2017) for program R (v. 3.6.1) (R Core Team, 2018). The initials for the first modeled count $I_{t=1,i}$ were drawn in log‐space from a normal distribution with the mean equal to the log of the first observation $y_{t=1,i}$ and a standard deviation of 1,000. We used vague normal priors with a mean of 0 and variance of 1,000 $\text{Normal}\left(0,1,000\right)$ for ${\bar{r}}_{i}$ and inverse gamma priors for both the process variance ($\sigma_{\eta }^{2}$) and estimable observation variance $\left(\sigma_{\text{est}}^{2}\right)$ of $\sigma^{2}∼\text{Inv‐Gamma(}0.001,0.001)$, which is approximately uniform on the log‐scale (Winker et al., 2018). We fit all models by running 4 Monte Carlo Markov chains (MCMC) for 500,000 iterations, with a burn‐in of 250,000 and a thinning rate of 5. Convergence was diagnosed by adopting maximal thresholds of $\hat{R}$ = 1.01 for Gelman–Rubin diagnostics (Gelman & Rubin, 1992). All models unambiguously converged. Unless otherwise specified, we report medians and 95% HPDI.

## 3 RESULTS

### 3.1 Global population

**FIGURE 2:**
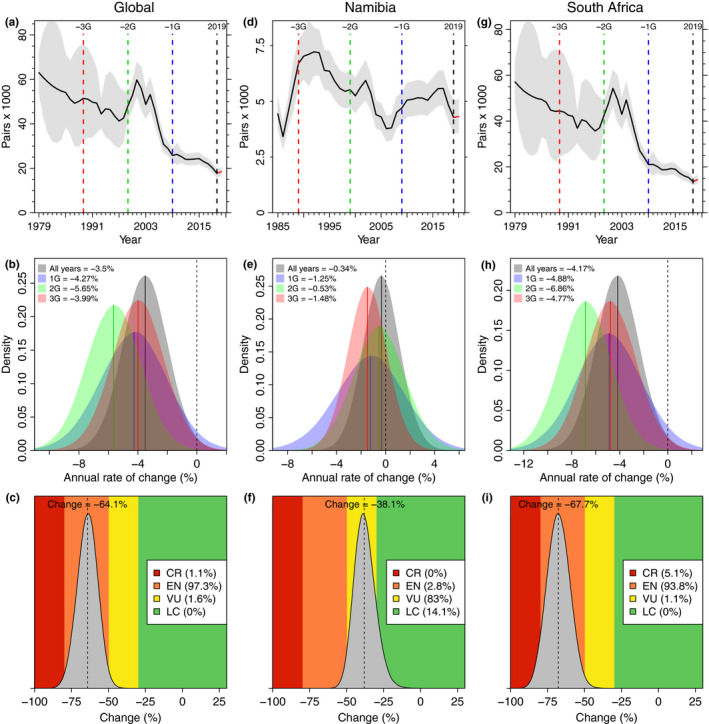
The decline in the global African penguin breeding population since 1979 (left, a, b, and c), in the Namibian population since 1985 (center, d, e, and f), and in the South African population since 1979 (right, g, h, and i). Top row (a, d, g): the JARA fitted median (black line) and 95% highest posterior density intervals (HPDI; gray polygon) for the population trend of African penguins based on nest counts from 22 colonies made between 1979 (1985 in Namibia) and 2019. The 10‐year generation lengths before 2019 are denoted by a blue dashed line (−1G, 2009), a green dashed line (−2G, 1999) and a red dashed line (−3G, 1989). Middle row (b, e, and h): the posterior medians (solid lines) and probability distributions (colored polygons) for the annual rate of population change (%) calculated from all the data (All years, in black and gray), from the last 10 years (1G; in blue), last 20 years (2G; in green), and last 30 years (3G; in red) shown relative to a stable population (% change = 0, black dashed line). Bottom row (c, f, and I): the median change (%, dashed line) in the breeding population of penguins globally (c) in Namibia (f) and in South Africa (I) over three generations (3G) or 30 years and corresponding posterior probability (gray polygon) for that change, overlaid on the IUCN thresholds for the Red List criterion A2 (LC—dark green, VU—yellow, EN—orange, CR—red)

Over the last 30 years (3G), the global African penguin population declined from ~51,500 pairs in 1989 to ~17,700 in 2019 (Figure 2a) at a median rate of change of  −4.0% (HPDI: −7.5 to −0.5%) per annum (Figure 2b). This corresponds to a 64.1% (51.0%–77.5%) decline, with 97.3% probability that the species meets the IUCN Red List classification of globally Endangered (EN) under criterion A2 (Figure 2c). The annual rate of change has remained around −4% since 1979 (All years = −3.5%, −6.5 to −0.5%), but peaked at −5.6% (−9.2 to −2.1%) over the last 20 years (2G; Figure 2b). For the global model, process error ($\sigma_{\eta }$) = 0.384 (0.356–0.412) and the total observation error ($\sigma_{\varepsilon }$) = 0.157 (0.151–0.178). The individual rates of change ($\bar{r}$) for each colony are in Table S1, Appendix 1.

### 3.2 Namibia—national status and trend

In Namibia, the African penguin population has fluctuated since 1985 (Figure 2d). Over the last 30 years (3G), however, the modeled population declined from ~6,700 pairs in 1989 to ~4,300 pairs in 2019 (Figure 2d), mostly as a result of declines at Ichaboe Island (Figure S2, Appendix 1). The median rate of change varied between −0.3 (−3.3 to +2.6) and − 1.5 (−4.6 to +1.7)% (Figure 2e) as the population initially increased, then decreased through the 1990s and first half of the 2000s to a low of ~3,800 pairs in 2006, before recovering somewhat from 2008 (Figure 2d). Applying the IUCN Red List criterion A2 at a national level in Namibia would yield a classification of Vulnerable (VU) with a probability of 83.0% and a median decline over 30 years (3G) of 38.1% (23.4%–51.0%, Figure 2f). Process error ($\sigma_{\eta }$) = 0.206 (0.161–0.257) and the total observation error ($\sigma_{\varepsilon }$) = 0.160 (0.152–0.197) for the Namibian model run.

### 3.3 South Africa—national status and trend

Aside from a period of recovery during the late 1990s and early 2000s, the population in South Africa decreased fairly consistently since 1979 (Figure 2g), with an annual rate of change of −4.8% (−9.0 to −0.6%) over the last 30 years (3G; Figure 2h). Because of that period of recovery, the rate of change was fastest over the last 20 years (2G) at −6.9% (−11.1 to −2.7%), but the population continued to decline at −4.9% (−10.3 to +0.6%) per annum over the last 10 years (1G; Figure 2h). Applying the IUCN Red List criterion A2 at a national level in South Africa would yield a classification of EN with a probability of 93.8% and a median decline over 30 years (3G) of 67.7% (52.9%–82.5%, Figure 2i). Process error ($\sigma_{\eta }$) = 0.418 (0.385–0.457) and the total observation error ($\sigma_{\varepsilon }$) = 0.158 (0.151–0.185) for the model run with only the South African data.

### 3.4 Regional trends within South Africa

**FIGURE 3:**
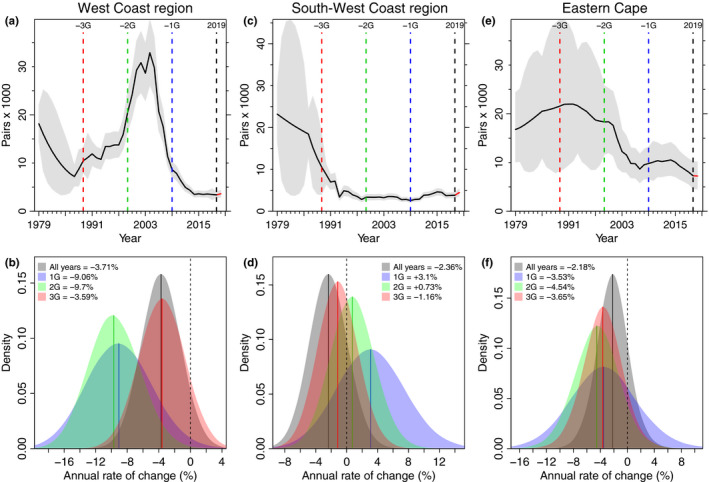
The change in the African penguin breeding population within the three regions of South Africa: the West Coast region (Western Cape colonies north of Cape Town; left, a and b), the South‐West Coast region (Western Cape colonies south and east of Cape Town, middle, c and d) and the Eastern Cape (right, e and f). (a, c, and e) The median (black line) and 95% HPDI (gray polygon) for the regional population trends of African penguins. The 10‐year generation lengths before 2019 are denoted by a blue dashed line (−1G, 2009), a green dashed line (−2G, 1999), and a red dashed line (−3G, 1989). (b, d and f) The posterior medians (solid lines) and probability distributions (colored polygons) for the annual rate of population change (%) calculated from all the data (1979 to 2019, All years, in black and gray), and from the last 10 years (1G; in blue), last 20 years (2G; in green), and last 30 years (3G; in red), shown relative to a stable population (% change = 0, black dashed line)

Within South Africa, the bulk of the recovery seen in the national trend (Figure 2g) resulted from growth in the population in the West Coast region (Figure 3a), mainly Dassen Island and Robben Island (Figure S1, Appendix 1). Again, in part because of that period of growth and recovery, the rate of change over the last 20 years (2G) has been substantial, at −9.7% (−15.9 to −3.3%, Figure 3b). However, unlike elsewhere, this rapid decline persisted in recent years; the rate of change at the colonies in the West Coast region over the last 10 years (1G) was −9.1% (−17.0 to −0.9%, Figure 3b). Overall, this regional population has declined by 68.7% (58.5%–77.3%) at an annual rate of change of −3.6% (−9.2 to +2.1%) per annum over the last 30 years. Moreover, there was little uncertainty in this decline; if the IUCN Red List criterion A2 was applied at a regional level, this subpopulation would qualify for an EN status with 99.7% probability (Figure S4, Appendix 1). Process error ($\sigma_{\eta }$) = 0.380 (0.339–0.428) and the total observation error ($\sigma_{\varepsilon }$) = 0.157 (0.151–0.182) for the West Coast regional model run.

The trend at colonies in the South‐West Coast region was initially dominated by a decline at Dyer Island, from ~23,000 pairs in 1979 to ~2,300 pairs in 1999 and ~1,060 pairs in 2019 (Figure S1, Appendix 1); thus the median rate of change since 1979 was −2.4% (−7.1 to +2.6%) overall and −1.2% (−6.3 to +4.0%) since 1989 (3G, Figure 3c). More recently, the decreases at Dyer Island were somewhat offset by the colonization and growth (since the 1980s) of the land‐based colonies at Simonstown and Stony Point to ~980 and ~1,750 pairs, respectively (Figure S1, Appendix 1). As these two colonies have come to dominate the population numbers in this region, so the annual rate of change has shifted from negative to positive, ending at +3.1% (−5.4 to +11.8%) in the last 10 years (1G; Figure 3d). However, these increases did not offset the ~90% decline of the population at Dyer Island (Figure 3c). Process error ($\sigma_{\eta }$) = 0.424 (0.367–0.496) and the total observation error ($\sigma_{\varepsilon }$) = 0.159 (0.151–0.197) for the South‐West Coast model.

In the Eastern Cape, the population has decreased fairly consistently since 1989 (Figure 3e) at rate of change varying from −3.5% (−13.5 to +6.6%) to −4.5% (−11.2 to +2.0%), and which has in general been slightly slower than the overall rate of change in South Africa (cf. Figure 3f with Figure 2h). Although this subpopulation has declined by 66.2% (35.6%–88.5%) over the last 30 years (3G; Figure S4, Appendix 1), it has come to represent a far greater proportion of the overall African penguin population in South Africa as a result of the substantial declines at Dyer Island and the colonies north of Cape Town (in particular Dassen Island). In 1979, the six Eastern Cape colonies contained 27% (19%–34%) of the total African penguin population. In 2019, they contained 41% (29%–52%). Process error ($\sigma_{\eta }$) = 0.267 (0.155–0.417) and the total observation error ($\sigma_{\varepsilon }$) = 0.377 (0.255–0.481) for the Eastern Cape model.

## 4 DISCUSSION

African penguin numbers declined steadily over the last three decades, resulting in a loss of almost 65% since 1989, and reached an historical low of ~17,700 pairs in 2019. Our results strongly support its classification as globally Endangered on the IUCN Red List and indicate a clear cause for concern for this species. However, the Bayesian state‐space models underpinning JARA allowed us to decompose this decline—particularly the variation in the annual rates of change—in both space and time, and to demonstrate robustly that the African penguin population has not decreased uniformly across its range. This variability has arisen for several reasons, including differences in the nature and severity of threats and local population dynamics. It follows, then, that there are different conservation management priorities for each subpopulation.

The Namibian population has declined at a slower rate than the other regional populations over the last three decades, with the rate of decline sufficient to warrant a Red List classification of Vulnerable under criterion A (Figure 2f). However, the Namibian penguin population had already declined by ~70% prior to the start of our dataset in 1986, coincident with the collapse of the Namibian sardine stocks in the 1970s (Crawford, 2007). The population also underwent a worrying decline to 3,800 pairs in 2006 before recovering slightly to 4,300 pairs by 2019. The penguin population in Namibia is likely now constrained at a low number by a scarcity of small pelagic fish (Roux et al., 2013; Watermeyer, Shannon, Roux, & Griffiths, 2008) and the birds' reliance on lower energy prey (Ludynia, Roux, Jones, Kemper, & Underhill, 2010). Monitoring of breeding colonies in Namibia is an ongoing priority, with an annual census of breeding pairs the minimum requirement to track trends in this population. A recent outbreak of avian influenza in some colonies in Namibia has shown the vulnerability of this population to stochastic events (Molini et al., 2020), the effects of which are exacerbated at low population levels (Lande, 1993).

The South African population recently declined at a much faster rate than the one in Namibia, resulting in a national and global classification of Endangered. Despite a small population recovery in the late 1990s and first half of the 2000s, driven mostly by increases in the West Coast region, there was a subsequent crash from the mid‐2000s onwards to an historical low in South Africa of ~13,600 pairs in 2019. The short‐lived population recovery and subsequent crash were associated with a concomitant boom and then decline in sardine and anchovy biomass (Crawford et al., 2011). This decline also coincided with an eastward displacement of a number of marine resources in South Africa (Blamey et al., 2015), including spawning adults of sardine and anchovy (Coetzee, van der Lingen, Hutchings, & Fairweather, 2008; Roy, van der Lingen, Coetzee, & Lutjeharms, 2007). These environmental changes combined with fishing pressure (Coetzee et al., 2008; Mhlongo, Yemane, Hendricks, & van der Lingen, 2015) to lower the availability of prey for seabirds breeding to the north of Cape Town (Crawford, Sydeman, Thompson, Sherley, & Makhado, 2019). This loss of their prey base underpinned the dramatic and unsustainable decline at almost 10% per year over the last 20 years at the West Coast colonies (Figure 3b). In contrast, penguin numbers in the South‐West Coast region have remained relatively stable at low levels over the last 30 years, principally supported by growth of the mainland colonies at Simonstown and Stony Point, which has somewhat offset the recent portion of the long‐term decline at Dyer Island (Figure S1). Meanwhile, the Eastern Cape region has experienced periods of relative stability followed by declines in the early 2000s and the late 2010s. Because the Eastern Cape population has declined at a slower rate than elsewhere in South Africa, the area has become increasingly important in terms of its relative contribution to the global population. At the same time, Algoa Bay has been identified as a marine transport hub, with permitted ship to ship bunkering taking place, and potentially as an Aquaculture Development Zone (Massie et al., 2019), increasing the risks of oil spills and other human impacts on the ecosystem (Pichegru, Nyengera, McInnes, & Pistorius, 2017; Ryan, Ludynia, & Pichegru, 2019). Since bunkering was permitted in Algoa Bay, for example, two bunkering‐related oil spills have taken place, in 2016 and 2019, oiling 220 African penguins (Ryan et al., 2019).

A lack of suitable prey, predominantly small pelagic fish, is believed to be the main driver for declines in African penguin numbers in South Africa over the last three decades (Crawford et al., 2011, 2019; Crawford, Sabarros, Fairweather, Underhill, & Wolfaardt, 2008; Sherley et al., 2017), with sporadic oiling events, habitat destruction, disturbance, and predation also contributing to the losses (Crawford et al., 2000; Makhado, Crawford, Waller, & Underhill, 2013; Pichegru, 2012; Weller et al., 2014). In 2013, the South African government put in place a Biodiversity Management Plan (BMP) for the African penguin (DEA, 2013). This plan aimed to halt the decline of the species and thereafter achieve the down listing of the species' conservation status. Although the plan did not achieve its aim, it provided a coordinated approach to penguin conservation and several key conservation interventions were initiated, or given greater credence, through this plan. One conservation intervention given increased importance in the BMP is the identification and protection of important foraging areas. Work along these lines has focused predominately on a 12‐year experiment, started in 2008, to investigate the effects of fishing closures around penguin breeding colonies. The experiment has shown some benefits to breeding penguins through a decrease in foraging effort and an increase in chick growth and condition when fishing was prohibited (Pichegru et al., 2012; Sherley et al., 2015, 2018) (although this has been contested, Butterworth, Plagányi, Robinson, Moosa, & Moor, 2015; Robinson, Butterworth, & Plagányi, 2015; Weller et al., 2016). The recent stability of breeding numbers at Simonstown (small pelagic fishing in False Bay has been prohibited since 1982, Penney, 1991) and Stony Point (which is surrounded by a small marine protected area) during a period when the populations at all the other South African colonies have declined also provides circumstantial evidence in support of protecting the key foraging areas used by breeders.

The initial identification of areas used by penguins during other parts of their life cycle such as pre‐ and post‐molt and during the first few years after fledging has also begun (Roberts, 2016; Sherley et al., 2017), but further work is required to determine the most appropriate mechanism to protect penguins during these vulnerable periods (Sherley et al., 2017). Additional spatial management of sardine and anchovy fishing effort, currently concentrated on the West Coast, will assist with addressing the mismatch between fish distribution and fishing effort (Coetzee et al., 2008; Grémillet et al., 2008). The hand‐rearing and release of chicks (Sherley, Waller, et al., 2014), and the creation of new breeding colonies have also been suggested as ways to mitigate the mismatch between penguin breeding colonies and fish distribution (DEA, 2013) and a pilot site to establish a colony is currently underway on the southern coast of South Africa. A revised BMP is being prepared with fewer, more threat‐focused actions, and will be implemented from 2020.

Our results highlight the dynamic nature of the decline of the African penguin population and have clarified the long‐term regional population trajectories. We identified an unsustainable decline of almost 10% per year at colonies to the north of Cape Town, the former geographic core of the species' breeding range. Our results reiterate the southward and eastward shift that has been observed in several marine species in South Africa (Jarre et al., 2015) and denote a change to a condition where colonies at the geographic edge of the species' range in the Eastern Cape now form the stronghold of the African penguin population. Marine taxa in the region are unable to move any further south, so these eastward shifts may be analogous to the poleward shifts seen in marine taxa elsewhere (Hastings et al., 2020). Accordingly, the Eastern Cape colonies should be viewed as a priority for conservation interventions, as should actions that could contribute to retaining viable breeding populations at the formerly large colonies in the West Coast region (Sherley et al., 2018). Finally, the robust JARA‐based Red List Assessments we used provide transparent estimates of uncertainty, accommodate nonlinearity in population trajectories, and account for observation heterogeneities (Sherley, Winker, et al., 2020). Thus, our approach has wide potential applicability to other studies of wildlife populations threatened with extinction.

## CONFLICT OF INTEREST

The authors declare no competing interests.

## AUTHOR CONTRIBUTIONS

**Richard B. Sherley:** Conceptualization (equal); Data curation (supporting); Formal analysis (lead); Funding acquisition (lead); Investigation (supporting); Methodology (equal); Project administration (lead); Resources (equal); Software (supporting); Validation (lead); Visualization (lead); Writing‐original draft (lead); Writing‐review & editing (lead). **Robert J. M. Crawford:** Data curation (equal); Investigation (equal); Writing‐original draft (supporting); Writing‐review & editing (supporting). **Andrew D. de Blocq:** Writing‐original draft (equal); Writing‐review & editing (supporting). **Bruce M. Dyer:** Investigation (equal); Writing‐review & editing (supporting). **Deon Geldenhuys:** Investigation (equal); Writing‐review & editing (supporting). **Christina Hagen:** Writing‐original draft (equal); Writing‐review & editing (supporting). **Jessica Kemper:** Data curation (equal); Investigation (equal); Writing‐review & editing (supporting). **Azwianewi B. Makhado:** Data curation (equal); Investigation (equal); Writing‐review & editing (supporting). **Lorien Pichegru:** Investigation (equal); Writing‐review & editing (supporting). **Desmond Tom:** Data curation (equal); Investigation (equal); Writing‐review & editing (supporting). **Leshia Upfold:** Investigation (equal); Writing‐review & editing (supporting). **Johan Visagie:** Investigation (equal); Writing‐review & editing (supporting). **Lauren J. Waller:** Investigation (equal); Writing‐review & editing (supporting). **Henning Winker:** Conceptualization (equal); Formal analysis (supporting); Methodology (equal); Resources (equal); Software (lead); Validation (equal); Visualization (supporting); Writing‐review & editing (supporting).

### OPEN RESEARCH BADGES

This article has been awarded Open Data, Open Materials Badges. All materials and data are publicly accessible via the Open Science Framework at https://doi.org/10.5061/dryad.vx0k6djp7 and https://github.com/henning‐winker/JARA.

## Supporting information

Supplementary Material

Figure S1

Figure S2

Figure S3

Figure S4

## Data Availability

The data underlying this article are available in the Dryad digital repository: https://doi.org/10.5061/dryad.vx0k6djp7 (Sherley, Crawford, et al., 2020).

## References

[ece36554-bib-0001] Auger‐Méthé, M. , Field, C. , Albertsen, C. M. , Derocher, A. E. , Lewis, M. A. , Jonsen, I. D. , & Flemming, J. M. (2016). State‐space models' dirty little secrets: Even simple linear Gaussian models can have estimation problems. Scientific Reports, 6, 26677 10.1038/srep26677 27220686 PMC4879567

[ece36554-bib-0002] Bird, J. P. , Martin, R. , Akçakaya, H. R. , Gilroy, J. , Burfield, I. J. , Garnett, S. T. , … Butchart, S. H. M. (2020). Generation lengths of the world's birds and their implications for extinction risk. Conservation Biology. 10.1111/cobi.13486 32058610

[ece36554-bib-0003] BirdLife International . (2000). Threatened birds of the world. Barcelona, Spain: Lynx Edicions.

[ece36554-bib-0004] Blamey, L. K. , Shannon, L. J. , Bolton, J. J. , Crawford, R. J. M. , Dufois, F. , Evers‐King, H. , … Winker, H. (2015). Ecosystem change in the southern Benguela and the underlying processes. Journal of Marine Systems, 144, 9–29. 10.1016/j.jmarsys.2014.11.006

[ece36554-bib-0005] Butterworth, D. S. , Plagányi, É. E. , Robinson, W. M. L. , Moosa, N. , & de Moor, C. L. (2015). Penguin modelling approach queried. Ecological Modelling, 316, 78–80. 10.1016/j.ecolmodel.2015.08.001

[ece36554-bib-0006] Che‐Castaldo, C. , Jenouvrier, S. , Youngflesh, C. , Shoemaker, K. T. , Humphries, G. , McDowall, P. , … Lynch, H. J. (2017). Pan‐Antarctic analysis aggregating spatial estimates of Adélie penguin abundance reveals robust dynamics despite stochastic noise. Nature Communications, 8, 832 10.1038/s41467-017-00890-0 PMC5635117 29018199

[ece36554-bib-0007] Coetzee, J. C. , van der Lingen, C. D. , Hutchings, L. , & Fairweather, T. P. (2008). Has the fishery contributed to a major shift in the distribution of South African sardine? ICES Journal of Marine Science, 65, 1676–1688. 10.1093/icesjms/fsn184

[ece36554-bib-0008] Crawford, R. J. M. (2007). Food, fishing and seabirds in the Benguela upwelling system. Journal of Ornithology, 148(S2), S253–S260. 10.1007/s10336-007-0228-z

[ece36554-bib-0009] Crawford, R. , Altwegg, R. , Barham, B. J. , Barham, P. J. , Durant, J. M. , Dyer, B. M. , … Whittington, P. A. (2011). Collapse of South Africa's penguins in the early 21st century. African Journal of Marine Science, 33(1), 139–156. 10.2989/1814232X.2011.572377

[ece36554-bib-0010] Crawford, R. J. M. , Davis, S. A. , Harding, R. T. , Jackson, L. F. , Leshoro, T. M. , Meÿer, M. A. , … Wolfaardt, A. C. (2000). Initial impact of the Treasure oil spill on seabirds off western South Africa. South African Journal of Marine Science, 22, 157–176. 10.2989/025776100784125645

[ece36554-bib-0011] Crawford, R. J. M. , Kemper, J. , & Underhill, L. G. (2013). African penguin *Spheniscus demersus* In Garcia‐Borboroglu P., & Boersma P. D. (Eds.), Penguins: Natural History and Conservation (pp. 211–231). Seattle, WA: University of Washington Press.

[ece36554-bib-0012] Crawford, R. J. M. , Makhado, A. B. , & Oosthuizen, W. H. (2018). Bottom‐up and top‐down control of the Benguela ecosystem's seabirds. Journal of Marine Systems, 188, 133–141. 10.1016/j.jmarsys.2017.04.004

[ece36554-bib-0013] Crawford, R. J. M. , Sabarros, P. , Fairweather, T. , Underhill, L. G. , & Wolfaardt, A. (2008). Implications for seabirds off South Africa of a long‐term change in the distribution of sardine. African Journal of Marine Science, 30, 177–184. 10.2989/AJMS.2008.30.1.18.468

[ece36554-bib-0014] Crawford, R. J. M. , Shannon, L. J. , & Whittington, P. A. (1999). Population dynamics of the African penguin *Spheniscus demersus* at Robben Island, South Africa. Marine Ornithology, 27, 139–147.

[ece36554-bib-0015] Crawford, R. J. M. , Sydeman, W. J. , Thompson, S. A. , Sherley, R. B. , & Makhado, A. B. (2019). Food habits of an endangered seabird indicate recent poor forage fish availability off western South Africa. ICES Journal of Marine Science, 76, 1344–1352. 10.1093/icesjms/fsz081

[ece36554-bib-0016] Crawford, R. J. M. , Underhill, L. G. , Upfold, L. , & Dyer, B. M. (2007). An altered carrying capacity of the Benguela upwelling ecosystem for African penguins (*Spheniscus demersus*). ICES Journal of Marine Science, 64, 570–576. 10.1093/icesjms/fsm009

[ece36554-bib-0017] Croxall, J. P. , Butchart, S. H. M. , Lascelles, B. , Stattersfield, A. J. , Sullivan, B. , Symes, A. , & Taylor, P. (2012). Seabird conservation status, threats and priority actions: A global assessment. Bird Conservation International, 22, 1–34. 10.1017/S0959270912000020

[ece36554-bib-0018] DEA . (2013). Biodiversity management plan for the African penguin (Spheniscus demersus). Department of Environmental Affairs, 68 pp. Retrieved from https://www.environment.gov.za/sites/default/files/gazetted_notices/africanpenguin_biodiversitymanagement_gn824_0.pdf

[ece36554-bib-0019] Dias, M. P. , Martin, R. , Pearmain, E. J. , Burfield, I. J. , Small, C. , Phillips, R. A. , … Croxall, J. P. (2019). Threats to seabirds: A global assessment. Biological Conservation, 237, 525–537. 10.1016/j.biocon.2019.06.033

[ece36554-bib-0020] Dufresne, D. (2004). The log‐normal approximation in financial and other computations. Advances in Applied Probability, 36, 747–773. 10.1239/aap/1093962232

[ece36554-bib-0021] Francis, R. I. C. C. , Hurst, R. J. , & Renwick, J. A. (2003). Quantifying annual variation in catchability for commercial and research fishing. Fisheries Bulletin, 101, 293–304.

[ece36554-bib-0022] Froese, R. , Demirel, N. , Coro, G. , Kleisner, K. M. , & Winker, H. (2017). Estimating fisheries reference points from catch and resilience. Fish and Fisheries, 18, 506–526. 10.1111/faf.12190

[ece36554-bib-0023] Frost, P. G. H. , Siegfried, W. R. , & Cooper, J. (1976). Conservation of the jackass penguin (*Spheniscus demersus* (L.)). Biological Conservation, 9, 79–99. 10.1016/0006-3207(76)90042-2

[ece36554-bib-0024] Gelman, A. , & Rubin, D. B. (1992). Inference from iterative simulation using multiple sequences. Statistical Science, 7, 457–511. 10.1214/ss/1177011136

[ece36554-bib-0025] Grémillet, D. , Lewis, S. , Drapeau, L. , van der Lingen, C. D. , Huggett, J. A. , Coetzee, J. C. , … Ryan, P. G. (2008). Spatial match–mismatch in the Benguela upwelling zone: Should we expect chlorophyll and sea‐surface temperature to predict marine predator distributions? Journal of Applied Ecology, 45, 610–621. 10.1111/j.1365-2664.2007.01447.x

[ece36554-bib-0026] Hastings, R. A. , Rutterford, L. A. , Freer, J. J. , Collins, R. A. , Simpson, S. D. , & Genner, M. J. (2020). Climate change drives poleward increases and equatorward declines in marine species. Current Biology, 30(8), 1572–1577.e2. 10.1016/j.cub.2020.02.043 32220327

[ece36554-bib-0027] IUCN Standards and Petitions Subcommittee . (2019). Guidelines for using the IUCN Red List Categories and Criteria. Version 14. Prepared by the Standards and Petitions Subcommittee. Gland, Switzerland and Cambridge, UK: IUCN Retrieved from http://www.iucnredlist.org/documents/RedListGuidelines.pdf

[ece36554-bib-0028] Jarre, A. , Hutchings, L. , Kirkman, S. P. , Kreiner, A. , Tchipalanga, P. C. M. , Kainge, P. , … Loeng, H. (2015). Synthesis: Climate effects on biodiversity, abundance and distribution of marine organisms in the Benguela. Fisheries Oceanography, 24, 122–149. 10.1111/fog.12086

[ece36554-bib-0029] Kellner, K. (2017). jagsUI: A Wrapper Around ‘rjags’ to Streamline ‘JAGS’ Analyses (R package version 1.4.9). Retrieved from https://CRAN.R‐project.org/package=jagsUI

[ece36554-bib-0030] Kemper, J. (2015). African penguin (Jackass penguin) *Spheniscus demersus* In Simmons R., Brown C., & Kemper J. (Eds.), Birds to watch in Namibia: Red, rare and endemic species (pp. 183–185). Windhoek, Namibia: Ministry of Environment and Tourism and Namibia Nature Foundation.

[ece36554-bib-0031] Kéry, M. , & Schaub, M. (2012). Bayesian population analysis using WinBUGS: A hierarchical perspective. Oxford, UK: Academic Press.

[ece36554-bib-0032] Lande, R. (1993). Risks of population extinction from demographic and environmental stochasticity and random catastrophes. American Naturalist, 142, 911–927. 10.1086/285580 29519140

[ece36554-bib-0033] Ludynia, K. , Roux, J.‐P. , Jones, R. , Kemper, J. , & Underhill, L. G. (2010). Surviving off junk: Low‐energy prey dominates the diet of African penguins *Spheniscus demersus* at Mercury Island, Namibia, between 1996 and 2009. African Journal of Marine Science, 32, 563–572. 10.2989/1814232X.2010.538151

[ece36554-bib-0034] Makhado, A. B. , Crawford, R. J. M. , Waller, L. J. , & Underhill, L. G. (2013). An assessment of the impact of predation by Cape fur seals *Arctocephalus pusillus pusillus* on seabirds at Dyer Island, South Africa. Ostrich, 84, 191–198. 10.2989/00306525.2013.863234

[ece36554-bib-0035] Massie, V. , Clark, B. , Hutchings, K. , Dawson, J. , Brown, E. , Wright, A. , & Laird, M. (2019). Proposed Sea‐based Aquaculture Development Zone in Algoa Bay, Eastern Cape –Final Basic Assessment Report in Terms of the National Environmental Management Act (107 of 1998). Report prepared for the Department of Agriculture, Forestry and Fisheries by Anchor Research and Monitoring (Pty) Ltd. Retrieved from https://anchorenvironmental.co.za/sites/default/files/2019‐10/Algoa%20BayADZ_FinalBAR_Anchor.pdf

[ece36554-bib-0036] Maunder, M. N. , & Piner, K. R. (2017). Dealing with data conflicts in statistical inference of population assessment models that integrate information from multiple diverse data sets. Fisheries Research, 192, 16–27. 10.1016/j.fishres.2016.04.022

[ece36554-bib-0037] Methot, R. D. , & Taylor, I. G. (2011). Adjusting for bias due to variability of estimated recruitments in fisheries assessment models. Canadian Journal of Fisheries and Aquatic Sciences, 68, 1744–1760.

[ece36554-bib-0038] Mhlongo, N. , Yemane, D. , Hendricks, M. , & van der Lingen, C. D. (2015). Have the spawning habitat preferences of anchovy (*Engraulis encrasicolus*) and sardine (*Sardinops sagax*) in the southern Benguela changed in recent years? Fisheries Oceanography, 24(S1), 1–14. 10.1111/fog.12061

[ece36554-bib-0039] Molini, U. , Aikukutu, G. , Roux, J.‐P. , Kemper, J. , Ntahonshikira, C. , Marruchella, G. , … Dundon, W. G. (2020). Avian Influenza H5N8 outbreak in African penguins (*Spheniscus demersus*), Namibia, 2019. Journal of Wildlife Diseases, 56, 214–218. 10.7589/2019-03-067 31483707

[ece36554-bib-0040] Paleczny, M. , Hammill, E. , Karpouzi, V. , & Pauly, D. (2015). Population trend of the world's monitored seabirds, 1950–2010. PLoS One, 10, e0129342 10.1371/journal.pone.0129342 26058068 PMC4461279

[ece36554-bib-0041] Penney, A. J. (1991). The interaction and impact of net and line‐fisheries in False Bay, South Africa. Transactions of the Royal Society of South Africa, 47, 663–681.

[ece36554-bib-0042] Pichegru, L. (2012). Increasing breeding success of an Endangered penguin: Artificial nests or culling predatory gulls? Bird Conservation International, 23, 296–308. 10.1017/S0959270912000135

[ece36554-bib-0043] Pichegru, L. , Nyengera, R. , McInnes, A. M. , & Pistorius, P. (2017). Avoidance of seismic survey activities by penguins. Scientific Reports, 7, 16305 10.1038/s41598-017-16569-x 29176687 PMC5701127

[ece36554-bib-0044] Pichegru, L. , Ryan, P. G. , Eeden, R. V. , Reid, T. , Grémillet, D. , & Wanless, R. (2012). Industrial fishing, no‐take zones and endangered penguins. Biological Conservation, 156, 117–125. 10.1016/j.biocon.2011.12.013

[ece36554-bib-0045] Plummer, M. (2003). JAGS: A program for analysis of Bayesian graphical models using Gibbs sampling In Hornik K., Leisch F., & Zeileis A. (Eds.), Proceedings of the Third International Workshop on Distributed Statistical Computing (DSC 2003). Retrieved from http://sourceforge.net/projects/mcmc‐jags/

[ece36554-bib-0046] R Core Team . (2018). R: A language and environment for statistical computing. Vienna, Austria: R Foundation for Statistical Computing Retrieved from http://www.r‐project.org/

[ece36554-bib-0047] Roberts, J. (2016). African penguin (*Spheniscus demersus*) distribution during the non‐breeding season: Preparation for, and recovery from, a moulting fast. Msc thesis. University of Cape Town.

[ece36554-bib-0048] Robinson, W. M. L. , Butterworth, D. S. , & Plagányi, É. E. (2015). Quantifying the projected impact of the South African sardine fishery on the Robben Island penguin colony. ICES Journal of Marine Science, 72, 1882–1883. 10.1093/icesjms/fsv035

[ece36554-bib-0049] Roux, J.‐P. , van der Lingen, C. D. , Gibbons, M. J. , Moroff, N. E. , Shannon, L. J. , Smith, A. D. M. , & Cury, P. M. (2013). Jellyfication of marine ecosystems as a likely consequence of overfishing small pelagic fishes: Lessons from the Benguela. Bulletin of Marine Science, 89, 249–284. 10.5343/bms.2011.1145

[ece36554-bib-0050] Roy, C. , van der Lingen, C. , Coetzee, J. , & Lutjeharms, J. (2007). Abrupt environmental shift associated with changes in the distribution of Cape anchovy *Engraulis encrasicolus* spawners in the southern Benguela. African Journal of Marine Science, 29, 309–319. 10.2989/AJMS.2007.29.3.1.331

[ece36554-bib-0051] Ryan, P. G. , Ludynia, K. , & Pichegru, L. (2019). Ships' risky fuel transfers are threatening African Penguins. The Conversation. Retrieved from https://theconversation.com/ships‐risky‐fuel‐transfers‐are‐threatening‐african‐penguins‐121575

[ece36554-bib-0052] Shannon, L. J. , & Crawford, R. J. M. (1999). Management of the African penguin *Spheniscus demersus* – insights from modelling. Marine Ornithology, 27, 119–128.

[ece36554-bib-0053] Shelton, P. A. , Crawford, R. J. M. , Cooper, J. , & Brooke, R. K. (1984). Distribution, population size and conservation of the jackass penguin *Spheniscus demersus* . South African Journal of Marine Science, 2, 217–257. 10.2989/02577618409504370

[ece36554-bib-0054] Sherley, R. B. , Abadi, F. , Ludynia, K. , Barham, B. J. , Clark, A. E. , & Altwegg, R. (2014). Age‐specific survival and movement among major African Penguin *Spheniscus demersus* colonies. Ibis, 156, 716–728. 10.1111/ibi.12189

[ece36554-bib-0055] Sherley, R. B. , Barham, B. J. , Barham, P. J. , Campbell, K. J. , Crawford, R. J. M. , Grigg, J. , … Votier, S. C. (2018). Bayesian inference reveals positive but subtle effects of experimental fishery closures on marine predator demographics. Proceedings of the Royal Society B: Biological Sciences, 285, 20172443 10.1098/rspb.2017.2443 PMC5805942 29343602

[ece36554-bib-0056] Sherley, R. B. , Crawford, R. J. M. , de Blocq, A. D. , Dyer, B. M. , Geldenhuys, D. , Hagen, C. , … Winker, H. (2020). Data from: The conservation status and population decline of the African penguin deconstructed in space and time. Dryad Digital Repository, 10.5061/dryad.vx0k6djp7 PMC7417240 32788996

[ece36554-bib-0057] Sherley, R. B. , Crawford, R. J. M. , Dyer, B. M. , Kemper, J. , Makhado, A. B. , Masotla, M. , … Winker, H. (2019). The status and conservation of Cape Gannets *Morus capensis* . Ostrich, 90, 335–346. 10.2989/00306525.2019.1684396

[ece36554-bib-0058] Sherley, R. B. , Ludynia, K. , Dyer, B. M. , Lamont, T. , Makhado, A. B. , Roux, J.‐P. , … Votier, S. C. (2017). Metapopulation tracking juvenile penguins reveals an ecosystem‐wide ecological trap. Current Biology, 27, 563–568. 10.1016/j.cub.2016.12.054 28190725

[ece36554-bib-0059] Sherley, R. B. , Waller, L. J. , Strauss, V. , Geldenhuys, D. , Underhill, L. G. , & Parsons, N. J. (2014). Hand‐rearing, release and survival of African penguin chicks abandoned before independence by moulting parents. PLoS One, 9(10), e110794 10.1371/journal.pone.0110794 25337698 PMC4206437

[ece36554-bib-0060] Sherley, R. B. , Winker, H. , Altwegg, R. , van der Lingen, C. D. , Votier, S. C. , & Crawford, R. J. M. (2015). Bottom‐up effects of a no‐take zone on endangered penguin demographics. Biology Letters, 11, 20150237 10.1098/rsbl.2015.0237 26156127 PMC4528441

[ece36554-bib-0061] Sherley, R. B. , Winker, H. , Rigby, C. L. , Kyne, P. M. , Pollom, R. , Pacoureau, N. , … Dulvy, N. K. (2020). Estimating IUCN Red List population reduction: JARA – a decision‐support tool applied to pelagic sharks. Conservation Letters, 13, e12688 10.1111/conl.12688

[ece36554-bib-0062] Watermeyer, K. E. , Shannon, L. J. , Roux, J.‐P. , & Griffiths, C. L. (2008). Changes in the trophic structure of the northern Benguela before and after the onset of industrial fishing. African Journal of Marine Science, 30, 383–403. 10.2989/AJMS.2008.30.2.12.562

[ece36554-bib-0063] Weller, F. , Cecchini, L.‐A. , Shannon, L. , Sherley, R. B. , Crawford, R. J. M. , Altwegg, R. , … Jarre, A. (2014). A system dynamics approach to modelling multiple drivers of the African penguin population on Robben Island, South Africa. Ecological Modelling, 277, 38–56. 10.1016/j.ecolmodel.2014.01.013

[ece36554-bib-0064] Weller, F. , Sherley, R. B. , Shannon, L. J. , Jarre, A. C. , Stewart, T. , Altwegg, R. , … Waller, L. J. (2016). Penguins' perilous conservation status calls for complementary approach based on sound ecological principles: Reply to Butterworth et al (2015). Ecological Modelling, 337, 1–3.

[ece36554-bib-0065] Whittington, P. A. (2002). Survival and movements of African penguins, especially after oiling. PhD thesis, University of Cape Town.

[ece36554-bib-0066] Whittington, P. A. , Klages, N. T. W. , Crawford, R. J. M. , Wolfaardt, A. C. , & Kemper, J. (2005). Age at first breeding of the African penguin. Ostrich, 76, 14–20. 10.2989/00306520509485468

[ece36554-bib-0067] Winker, H. , Carvalho, F. , & Kapur, M. (2018). JABBA: Just Another Bayesian Biomass Assessment. Fisheries Research, 204, 275–288. 10.1016/j.fishres.2018.03.010

[ece36554-bib-0068] Winker, H. , Pacoureau, N. , & Sherley, R. B. (2020). JARA: ‘Just Another Red‐List Assessment’. bioRxiv, 672899 10.1101/672899

