# Supplementary material for: The conservation status and population decline of the African penguin deconstructed in space and time
Source: Ecol Evol. 2020 Jul 9;10(15):8506–16. doi: 10.1002/ece3.6554 (PMC7417240; doi:10.1002/ece3.6554)
Supplement: Supplementary file 1 — Supplementary Material [file ECE3-10-8506-s005.docx]

**Appendix 1**

**
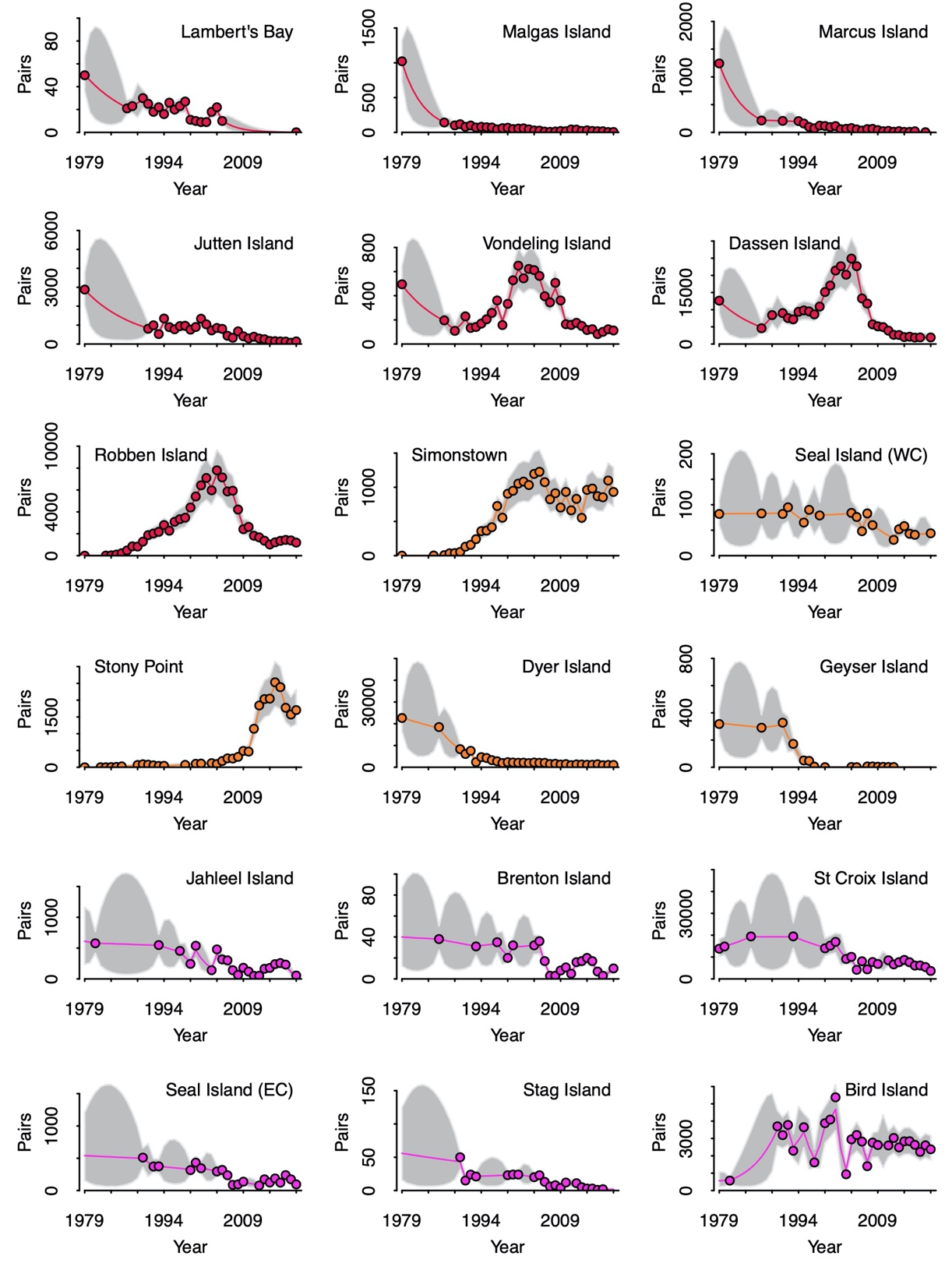
**

**Figure S1.** Bayesian state-space model fits (coloured lines) and 95% highest posterior density intervals (HPDI; grey polygons) from Just Another Red List Assessment (JARA) to population counts (points) made between 1979 and 2019 at 18 of the 19 known colonies in South Africa at which African penguins have bred for more than 5 years during that time frame. The colours denote the region to which each colony belongs: West Coast region (dark red, Lambert’s Bay to Robben Island), the South-West Coast region (orange, Simonstown to Geyser Island) and the Eastern Cape (purple, Jahleel Island to Bird Island).

**
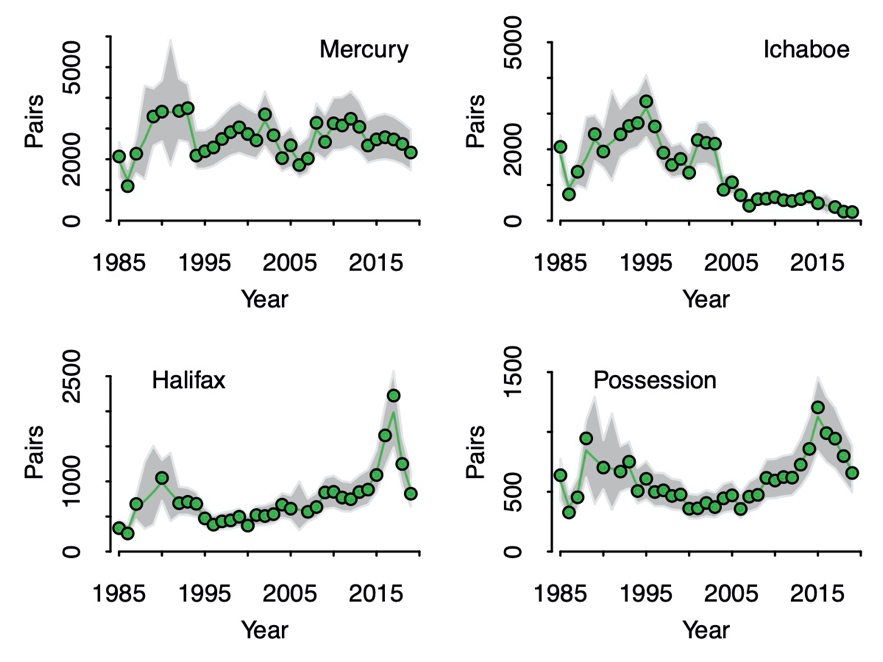
**

**Figure S2.** Bayesian state-space model fits (lines) from Just Another Red List Assessment (JARA) to population counts (points) made between 1985 and 2019 at the four breeding colonies in Namibia that contained ~95% of the Namibian African penguin population since 1985 (Kemper, 2015).


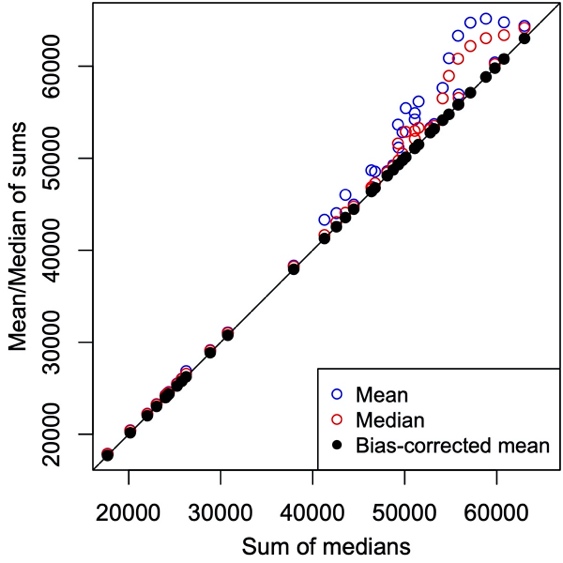


**Figure S3.** The relationship between the sum of the fitted population estimates (sum of the median of each individual posterior distribution) from all 22 African penguin breeding colonies in the global population model run and the mean (blue open circles), median (red open circles) and the bias-corrected mean (closed black points) of the sum of all 22 posterior distributions. The black line shows a 1-1 (unbiased) relationship. The difference between each point and the 1-1 line is the bias associated with summing log-normal distributions (Dufresne, 2004; Methot & Taylor, 2011; Che-Castaldo et al., 2017).


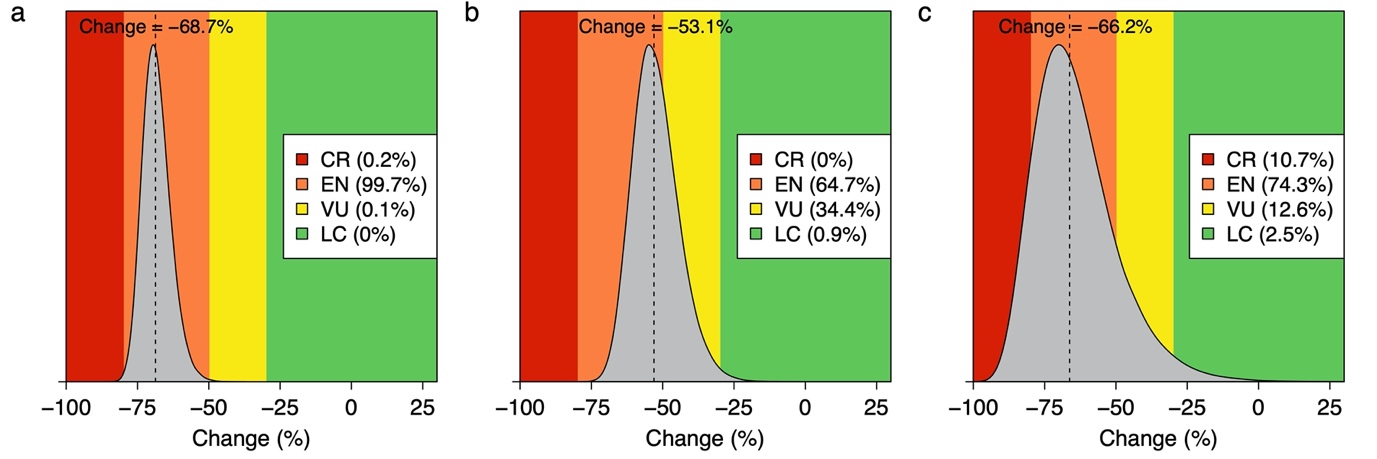


**Figure S4.** The median change (%, dashed line) in the breeding population of penguins in (a) the West Coast region of South Africa, (b) the South-West Coast region of South Africa, and (c) the Eastern Cape province of South Africa over three generations (3G), or 30 years, and corresponding posterior probability (grey polygon) for that change, overlaid on the IUCN Red List category thresholds for the Red List criteria A2 (LC—dark green, VU—yellow, EN—orange, CR—red).

**Table S1.** Parameter estimates from the Global Population JARA model run. The posterior median, standard deviation (SD) and 95% highest posterior density interval (HPDI) is show for the log population growth rate ($\bar{r}$) at each colony, the process error, estimable observation error and total observation error (all on the standard deviation scale).

| Parameter | Median | SD | 95% HPDI |
| --- | --- | --- | --- |
| Lambert’s Bay ($\bar{r}$) | −0.081 | 0.061 | −0.201–0.038 |
| Malgas Island ($\bar{r}$) | −0.055 | 0.061 | −0.174–0.066 |
| Marcus Island ($\bar{r}$) | −0.070 | 0.062 | −0.193–0.050 |
| Jutten Island ($\bar{r}$) | −0.004 | 0.061 | −0.124–0.116 |
| Vondeling Island ($\bar{r}$) | 0.037 | 0.061 | −0.082–0.157 |
| Dassen Island ($\bar{r}$) | 0.027 | 0.061 | −0.093–0.146 |
| Robben Island ($\bar{r}$) | 0.307 | 0.062 | 0.187–0.428 |
| Simonstown ($\bar{r}$) | 0.303 | 0.061 | 0.184–0.424 |
| Seal Island (False Bay) ($\bar{r}$) | 0.058 | 0.061 | −0.060–0.180 |
| Stony Point ($\bar{r}$) | 0.316 | 0.061 | 0.196–0.436 |
| Dyer Island ($\bar{r}$) | −0.003 | 0.061 | −0.124–0.116 |
| Geyser Island ($\bar{r}$) | −0.081 | 0.068 | −0.213–0.052 |
| Jahleel Island ($\bar{r}$) | 0.016 | 0.062 | −0.105–0.137 |
| Brenton Island ($\bar{r}$) | 0.037 | 0.062 | −0.085–0.158 |
| St. Croix Island ($\bar{r}$) | 0.041 | 0.061 | −0.079–0.161 |
| Seal Island (Algoa Bay) ($\bar{r}$) | 0.032 | 0.062 | −0.089–0.155 |
| Stag Island ($\bar{r}$) | −0.016 | 0.064 | −0.143–0.108 |
| Bird Island ($\bar{r}$) | 0.110 | 0.062 | −0.010–0.232 |
| Mercury Island ($\bar{r}$) | 0.076 | 0.062 | −0.047–0.197 |
| Ichaboe Island ($\bar{r}$) | 0.019 | 0.062 | −0.104–0.140 |
| Halifax Island ($\bar{r}$) | 0.071 | 0.062 | −0.049–0.194 |
| Possession Island ($\bar{r}$) | 0.076 | 0.062 | −0.046–0.198 |
| Process error ($\sigma_{\eta}$) | 0.384 | 0.014 | 0.355–0.411 |
| Estimable observation error ($\sigma_{est}$) | 0.045 | 0.020 | 0.017–0.088 |
| Total observation error ($\sigma_{\varepsilon}$) | 0.157 | 0.007 | 0.151–0.173 |
