# Supplementary figures and images for: The conservation status and population decline of the African penguin deconstructed in space and time
Source: Ecol Evol. 2020 Jul 9;10(15):8506–16. doi: 10.1002/ece3.6554 (PMC7417240; doi:10.1002/ece3.6554)

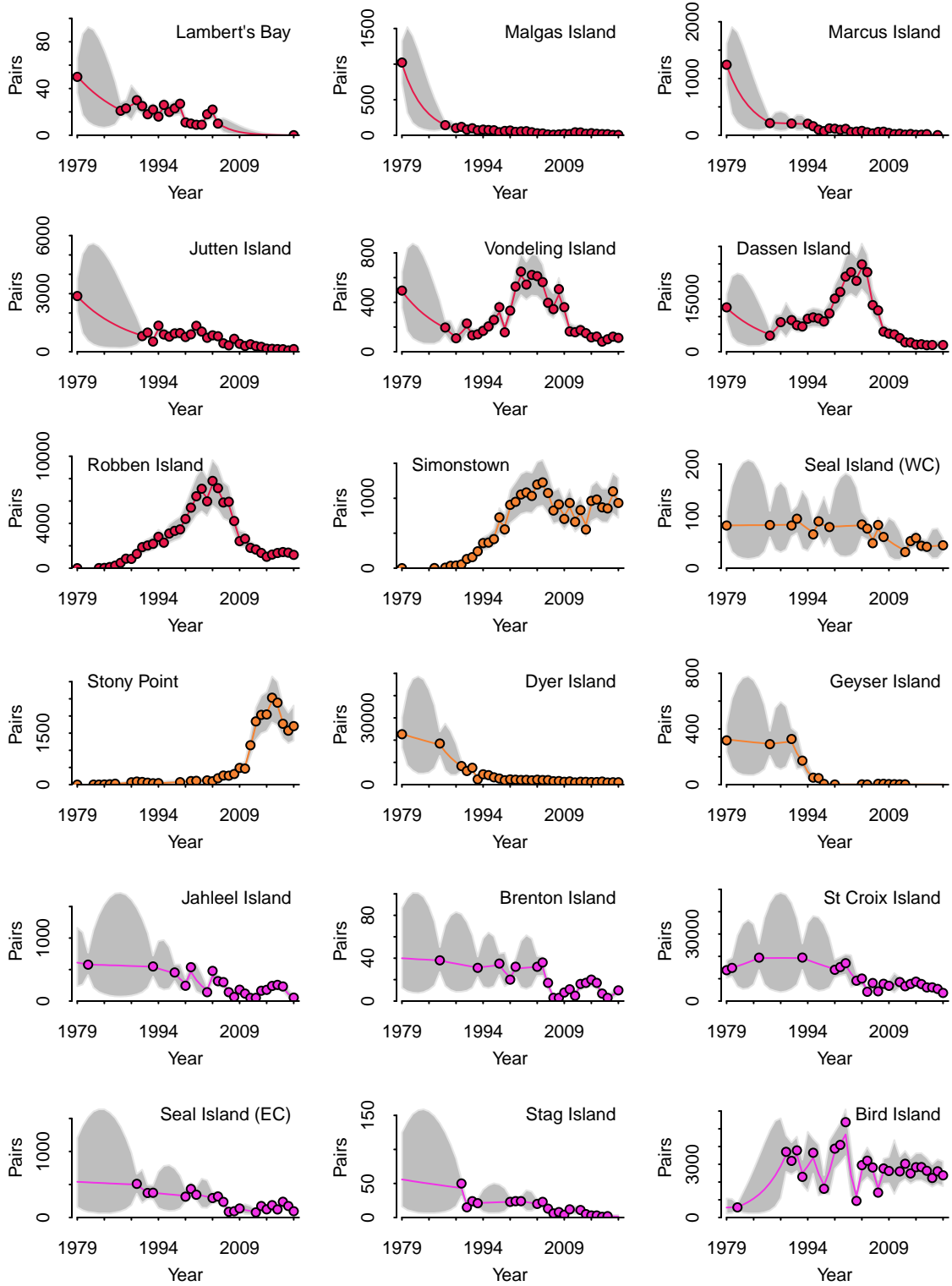

Supplement: Supplementary file 2 — Figure S1 [file ECE3-10-8506-s001.pdf]

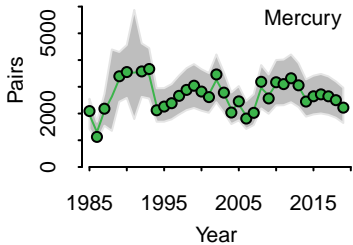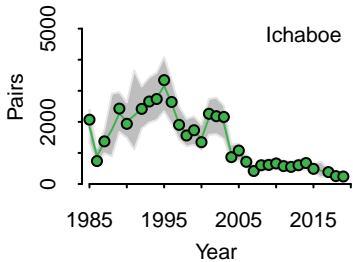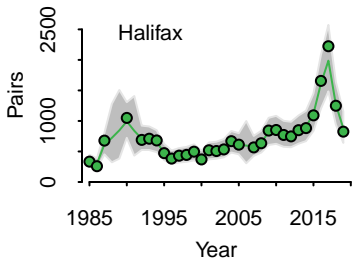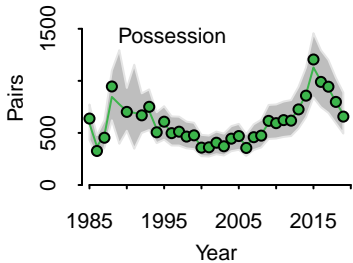

Supplement: Supplementary file 3 — Figure S2 [file ECE3-10-8506-s002.pdf]

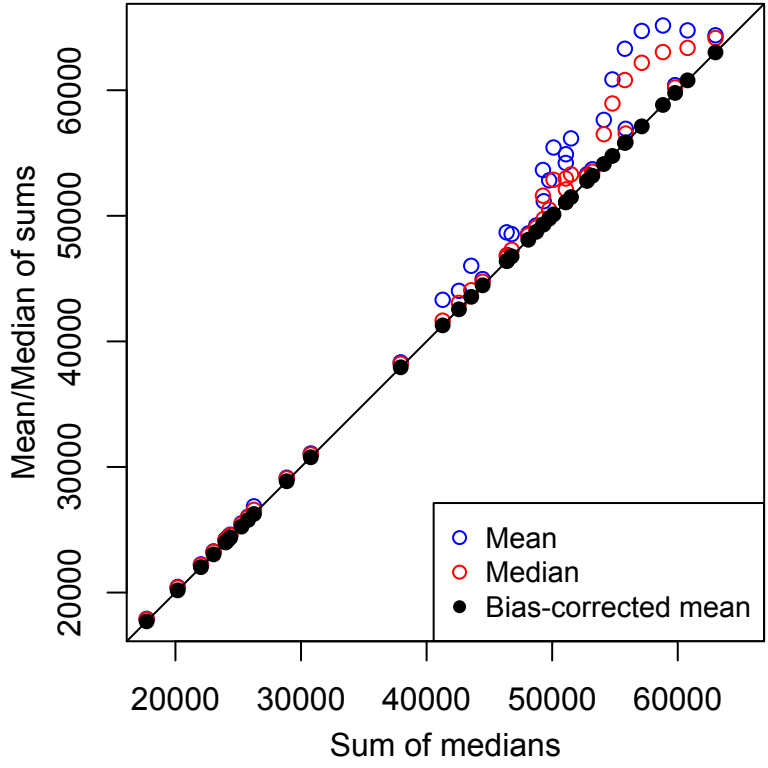

Supplement: Supplementary file 4 — Figure S3 [file ECE3-10-8506-s003.pdf]

a

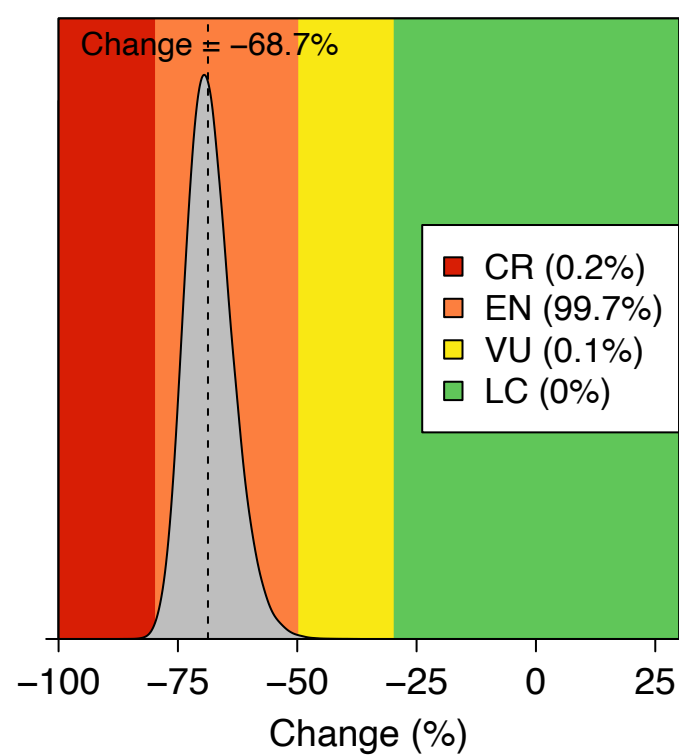

b

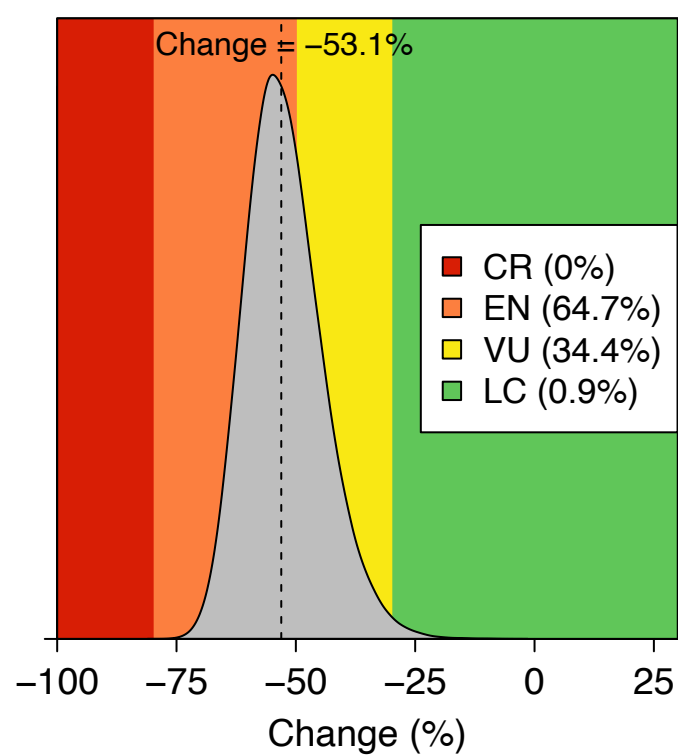

c

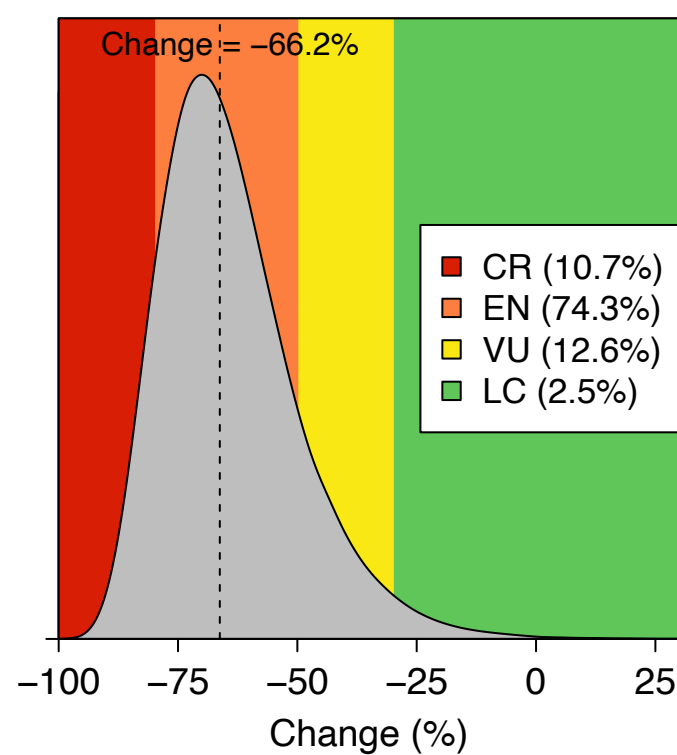

Supplement: Supplementary file 5 — Figure S4 [file ECE3-10-8506-s004.pdf]
